# A systematic review of the evidence of how hospitals capture financial benefits of process improvement and the impact on hospital financial performance

**DOI:** 10.1186/s12913-023-09258-1

**Published:** 2023-03-10

**Authors:** Jane Evans, Sandra G. Leggat, Danny Samson

**Affiliations:** 1 Improvement and Experience, St. Vincent’s Health Australia, Level 5, 340 Albert Road, East, Melbourne, Victoria 3002 Australia; 2 Health Services Management, School of Psychology and Public Health, La Trobe University, Bundoora, Victoria 3086 Australia; 3 Public Health & Tropical Medicine, James Cook University, Townsville, QLD 4811 Australia; 4 Department of Management and Marketing, University of Melbourne, 10Th Floor, 198 Berkeley St, Carlton, VIC 3010 Australia

**Keywords:** Waste, Value-based healthcare, Process improvement, Lean, Healthcare, Hospitals, Financial outcomes

## Abstract

**Background:**

Governments, funders and hospital managers around the world are looking for ways to address the continual growth in expenditure by reducing the level of waste in the healthcare delivery system and improving the value of care provided to patients. Process improvement methods are applied to increase high value care, reduce low value care and remove waste from care processes. The purpose of this study is to review the literature to identify the methods used by hospitals to measure and capture financial benefits from PI initiatives to identify best practice. The review also pursues the way hospitals collate these benefits at the enterprise level to achieve improved financial performance.

**Methods:**

A systematic review was undertaken in line with the PRISMA process and employed qualitative research methods. Databases searched were Medline, Cochrane Library, Cumulative Index to Nursing and Allied Health Literature (CINHAL), Web of Science and SCOPUS. The initial search was conducted in in July 2021 with a follow up search conducted in February 2023 using the same search terms and databases to identify additional studies published in the intervening period. The search terms were identified through the PICO (Participants, Interventions, Comparisons and Outcomes) method.

**Results:**

Seven papers were identified that reported reduction in care process waste or improvement of the value of care using an evidence-based PI approach and included financial benefits analysis. Positive financial impact was measured for the PI initiatives but none of the studies reported how these financial benefits were captured or applied at the enterprise level. Three of the studies suggested that sophisticated cost accounting systems were required to enable this.

**Conclusion:**

The study demonstrates the paucity of literature in the field of PI and financial benefits measurement in healthcare. Where financial benefits are documented, they vary in terms of cost inclusions and the ‘level’ at which the costs were measured. Further research on best practice financial measurement methods is needed to enable other hospitals to measure and capture financial benefits arising from their PI programs.

## Introduction

Throughout the world, annual spending on the provision of healthcare services continues to grow as a percentage of Gross Domestic Product (GDP). Literature from across the globe is consistent in estimating that one third of the money spent on the delivery of healthcare is waste [1, 2]. Waste is defined “….*as any activity or resource in an organization that does not add value to an external customer.” *([3] p. 2)*.*

Hospitals account for around 40% of overall healthcare costs. The acute hospital setting is the greatest contributor to these costs and therefore provides the principal opportunity to reduce global healthcare expenditure [4]. Moraros et al. ([5] p. 150) suggest that hospitals should focus on “*….. ‘removing waste’ in order to ‘add value’….”.* Porter ([6] p. 2477) defines value in healthcare as “…. *patient outcomes (quality and experience) achieved per dollar spent….”* introducing the connection between the care being provided and its outcomes, with the cost of that care. Bringing these two narratives together suggests that by removing waste and non-value adding care we can improve value, which according to the definition above, will have an associated financial impact.

In seeking to quantify the cost of waste in hospitals, Shrank et al. [7] calculated that the cost of waste in the health system of the United States of America (US) is between $760 billion and $935 billion US dollars (USD) per annum, or 25% of US overall healthcare spending. Of this, they estimate that there is a $200 billion opportunity to remove waste and reduce healthcare spending. Duckett et al. [2] calculate that Australia has a $1 billion Australian dollar (AUD) opportunity to reduce the cost of hospital care and recommends actions for governments and hospitals to take to address this cost [2]. Similar dollar calculations from other countries are not readily available in the literature however there is congruence in the estimated percentage of costs that may be characterised as waste and non-value adding [1]. In summary, there is a significant opportunity for hospitals to reduce expenditure on activities that do not add value, improve their bottom line, and potentially impact overall healthcare expenditure.

Process improvement methodologies in all forms, are the mechanism by which hospitals can control increasing costs through attention to waste and non-value adding care [2, 8–12]. Narayanan et al. ([13] p. 2) observe that PI “…… *may improve both cost and revenue performance, and consequently, bottom-line financial performance….”* Process improvement (PI) methods have different labels such as Lean, Process Redesign or Reengineering and Continuous Improvement, but all have a common aim. That is, to improve hospital processes through the removal of waste, the reduction of variation in processes and to increase the provision of value-based care [5, 14]. The intention of PI should not be approached as an exercise in cutting costs. Rather, financial benefits will arise from PI activity alongside other benefits such as improved patient outcomes and experience [4].

Consequently, to address non-value adding expenditure hospital managers should look to PI to remove and reduce waste in the processes of care. They must also focus on increasing the value of the care through the provision of evidence-based (high value) clinical care and removal of low value care. In this paper, the phrases "removing low value care" and "moving toward higher value care” are intended as complementary rather than synonymous as it is possible to have one without the other – although doing both at the same time is ideal. In moving towards increasing the value of care provided, managers need to be able to measure and realize the financial impact, both positive and negative [15]. In realising the financial impact of individual PI initiatives, consideration of how these are collectively amassed at the enterprise, company, or organization level and then ultimately to the health system level is important if we want to impact overall healthcare spending.

The purpose of this study is to systematically review the literature to identify methods by which hospitals measure and capture financial benefits from PI initiatives to identify best practice. The review also pursues the way hospitals collate these benefits at the enterprise level to achieve improved financial performance. The term ‘enterprise’ has been used throughout the paper to reference the hospital at the whole of organization level. The findings of this review are targeted at hospital administrators, PI experts, policy makers and hospital funding bodies. No study has systematically synthesized evidence in the literature on this topic. To this end, we addressed the following research questions:

- What methods do hospitals use to measure and realize the financial benefits of PI activities?
- Do hospitals collate financial benefits from PI programs at the hospital enterprise level?

The answer to these two questions is important for the following reasons. If hospitals are not able to measure and realize financial benefits of PI that are aimed at reducing non-value adding activity and low value care, and then collate these financial gains and apply them at the enterprise level, we must look to other means to impact the healthcare expenditure conundrum. We must start with the literature to understand current practice and how this can be used to guide other hospitals to undertake similar action.

## Methods

### Search

A systematic review of the healthcare services literature was undertaken in line with the Preferred Reporting Items for Systematic Reviews and Meta-analyses (PRISMA) Statement [16]. Databases searched were Medline, Cochrane Library, Cumulative Index to Nursing and Allied Health Literature (CINHAL), Web of Science and SCOPUS. The initial search was conducted in in July 2021 with a follow up search conducted in February 2023 using the same search terms and databases to identify additional studies published in the intervening period.

Five databases were selected for this study that cover a large range of journals focused on healthcare and hospitals, but also include research on hospital operations and management and health policy. The search terms were identified through the PICO (Participants, Interventions, Comparisons and Outcomes) method resulting in three categories or concepts being identified. Data was collected through searching the following key words “quality improvement” OR “Total Quality Management” OR “process improvement” OR “Lean” AND “hospitals” OR “healthcare” OR “healthcare management” OR “hospital operations” AND “healthcare costs” OR “cost benefit analysis” OR “efficiency”, OR “financial performance.” The search strategy was adapted for each database as necessary. Table 1 shows the complete search strategy.

**Table 1:** Search strategy

Concept	Search strategy
Process Improvement	“quality improvement” OR “Total Quality Management” OR “process improvement” OR “Lean”
AND hospital	“hospitals” OR “healthcare” OR “healthcare management” OR “hospital operations”
AND financial impact	“healthcare costs” OR “cost benefit analysis” OR “efficiency”, OR “financial performance.”

With several known publications describing the success of a hospital’s application of PI on financial outcomes not appearing in these database searches, a further search of grey literature was conducted using Google Scholar, using the same search terms. In addition, reference searches of identified articles and additional targeted searches based on research team input and knowledge was completed.

### Eligibility criteria and study selection

The articles from the search were exported to Covidence, duplicates were removed and three rounds of screening were undertaken. The first round of screening was undertaken by one reviewer (J.E.) and incorporated a review of the titles and key words of the articles identified. Inclusion criteria to proceed to the second round of screening were: English language publication; hospital setting; improvement methodology identified; and financial benefits or cost outcomes mentioned. Articles were included only if at least two of the inclusion criteria were present. Papers that were opinions, narratives or literature reviews were also excluded as the review is aimed at identification of hospital practice.

Second round screening was conducted by one reviewer (J.E.) incorporating a review of the abstract with inclusion criteria expanded to include: clear description of a PI methodology used; hospital setting; and financial outcomes of the PI activities defined. Publications from the year 2000 onwards (to the date of the search) were included because Lean or PI methods were not well applied in hospitals prior to this time. In addition, the focus on cost or expenditure in hospital has been of increasing concern over the last 20 years with findings from studies prior to this likely to be superseded. Articles were excluded if the research was undertaken at a non-hospital setting such as primary care or community setting, the PI methodology was absent, or financial benefits calculations were not included.

The third round of screening of the full text of the remaining articles was undertaken by two reviewers independently (J.E. and S.L.) seeking to identify research in a hospital setting with detail on the PI methodology utilized and specific financial benefits calculations undertaken included in the paper.

### Data extraction and analysis

A data extraction table was developed to enable reviewers to summarize the key characteristics of each study. The table headings are: general study descriptions such as hospital name and country; the timeframe; the scope or focus area of the PI activity; the PI methodology employed; and financial benefits analysis. These headings were pre-emptively defined by the two screening reviewers prior to undertaking the third round of screening to support the identification of studies that could contribute to answering the research questions. Following confirmation that the inclusion criteria were met, the data were organized to answer the research questions.

## Results

### Study selection and PRISMA flow diagram

**Fig. 1:**
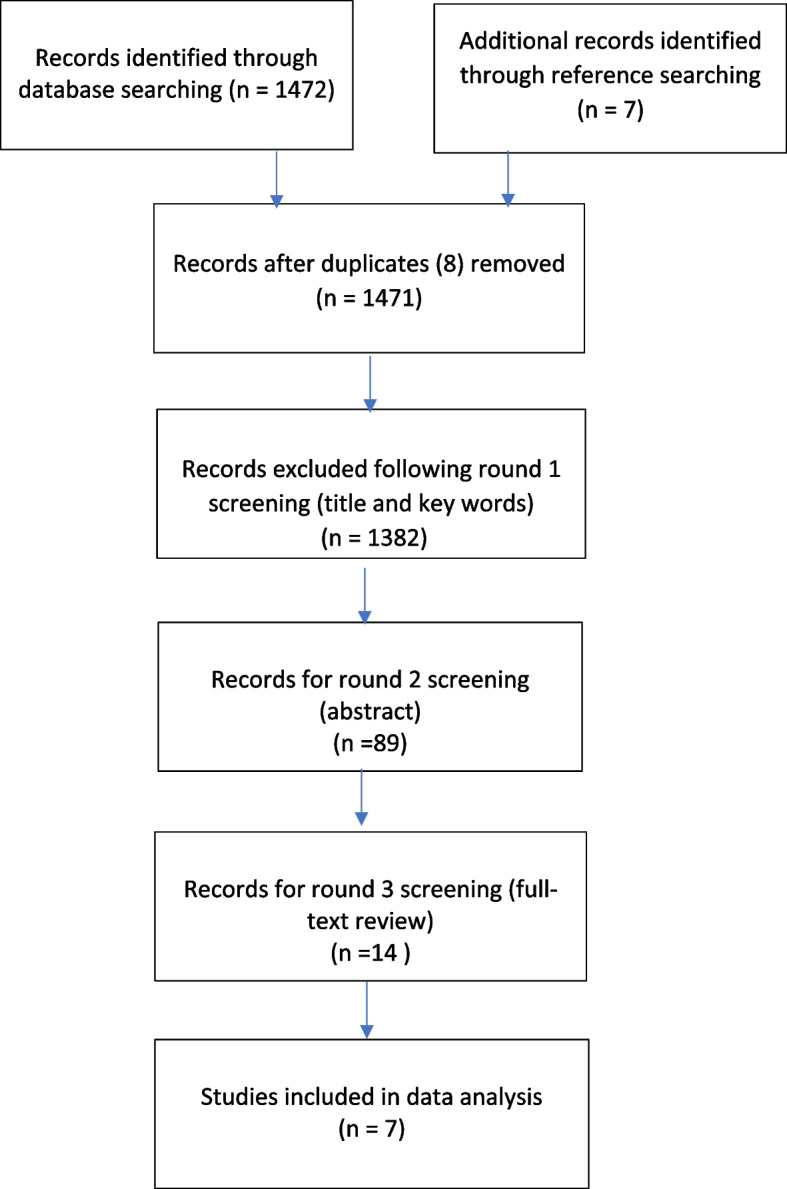
Flowchart of search results and record selection

A combined total of 1472 references were identified for screening after the database searches in July 2021 and update in February 2023. An additional 7 records were identified from reference searching resulting in 1471 studies to be screened against title and key words after 8 duplicates were removed (See Fig. 1).

Of these, 1382 studies were excluded because they did not mention at least two of the inclusion criteria in the title or key words leaving 89 studies for abstract assessment. A further 75 studies were excluded during round 2 screening as they were in a non-hospital setting, did not mention whether PI had been used, or had limited or no information about cost or financial impact calculation methods at either the project or enterprise level. A number were also excluded as they were published earlier than 2000 or were opinion, narrative or literature reviews. The remaining 14 papers underwent the third and final round of screening where independent review of the full text of the papers was undertaken by two reviewers. Of these, papers that did not explicitly describe all the three search field elements of: hospitals, PI methodology and financial information were omitted. In addition, papers were excluded where there was lack of information on the financial benefits and cost calculations meaning the validity of the financial outcome claim could not be tested despite assertions of positive financial outcomes.

The reviewers concurred that seven studies identified from the search were to be included in the analysis. The GRADE approach to rating the quality of evidence included in a systematic review has been employed to reflect the confidence in the findings and recommendations where four categories are defined that may be applied to a body of evidence rather than to individual studies: high; moderate; low; and very low [17].

### General paper characteristics

Four of the studies were set in hospitals in the U.S., one in Spain, one in Canadian and one in United Arab Emirates. Table 2 provides a summary of the key characteristics of papers included in this study.

**Table 2:** Summary of key characteristics of papers

Citation	Hospital	Date of study	Patient population and study focus area	PI methodology specified	Financial benefits achieved
Nimeri, A. A., Bautista, J., & Philip, R. (2019). Reducing healthcare costs using ACS NSQIP-driven quality improvement projects: a success story from Sheikh Khalifa Medical City (SKMC). World journal of surgery, 43(2), 331–338	Sheikh Khalifa Medical City’s surgical services Abu Dhabi, UAE	2009–15	Reduction in 4 Hospital Acquired Complications for all surgical patients during the study period	Targeted Quality Improvement	1. VTE reduction from 7/1646 to 26/ 7196, preventing 12 @ $28,000 for savings of $336,000; 2. UTI prevented 56 ($728,000) and SSI prevented 12 ($336,000); 3. UI prevented 4 ($84,000) and Vent > 48 h occurrences prevented was 7 ($196,000)
Chatfield SC, Volpicelli FM, Adler NM, et al. Bending the cost curve: time series analysis of a value transformation programme at an academic medical centre BMJ Qual Saf 2019;28:449–458	Tish Hospital NYU Langone Health, New York, United States	Baseline 2011–14; Post 2014–17	74 initiatives in acute clinical care services during study period	Standardised process improvement model	Fully adjusted total variable direct costs per case declined an average of 0.22% per month relative to baseline trends; net overall reduction in costs of 7.7% by the end of the study period relative to the expected cost without intervention
Lee, V. S., Kawamoto, K., Hess, R., Park, C., Young, J., Hunter, C., Pendleton, R. C. (2016). Implementation of a Value-Driven Outcomes Program to Identify High Variability in Clinical Costs and Outcomes and Association With Reduced Cost and Improved Quality. *JAMA, 316*(10), 1061–1072	University of Utah Health Care Utah, United States	July 1, 2014, to June 30, 2015	3 Clinical care projects: total hip and knee joint replacement; hospitalist laboratory utilization; and management of sepsis	Multiple PI methods specified including care process redesign, continuous improvement and process improvement and standardization	Mean direct costs were 7% lower in the implementation year (95%CI, 3%-11%;*P* < .001) and 11% lower in the post implementation year (95%CI, 7%-14%; *P* < .001)
Gabow, P. A. (2014). The Lean Prescription: Powerful Medicine for Our Ailing Healthcare System (1st ed.): OR: Productivity Press	Denver Health Colorado, United States	2005–2012	Whole of hospital – all patients	Lean	Almost $200 million of financial benefit over seven years with $50 million in 2012 alone
Barnas, K. (2011) ThedaCare’s Business Performance System: Sustaining Continuous Daily Improvement Through Hospital Management in a Lean Environment. The Joint Commission Journal on Quality and Patient Safety, 37( 9), 387–399	ThedaCare, Wisconsin, United States	2003–2008	Whole of hospitals	Lean	11 (48%) of the 23 financial stewardship drivers in 2010 compared to 2009 Specific improvement projects: The Collaborative Care Delivery Model: average decrease in cost per case of 22%. The Staffing to Patient Demand $895,000 salary savings Radiation Oncology Value Stream increased gross revenue by 24%,
Vincent Martinez Ibañez, Anna Ochoa de Echagüen, Antonio Campos & Soledad Romea (2021) Creating efficient professional healthcare organizations, International Journal of Healthcare Management, https://doi.org/10.1080/20479700.2020.1870347	University hospital Catalonia, Spain	2015–2018	Five processes or value streams: surgery; hospitalization; outpatient care; intensive care; and emergencies	Lean and Design Thinking	Financial results from the surgery stream only were reported to be a saving of 8,563,817 € through reducing waste and reordering surgical schedule and a revenue of increase of 600,044 € due to increasing surgical activity
Khowaja AR, Willms AJ, Krause C, Carriere S, Ridout B, Kennedy C, et al. The Return on Investment of a Province-Wide Quality Improvement Initiative for Reducing In-Hospital Sepsis Rates and Mortality in British Columbia, Canada. Critical Care Medicine. 2022;50(4):e340-e50	36 hospitals within British Columbia (excluding Quebec and three territories)	2014—2018	Retrospective data for all in-hospital sepsis cases and sepsis mortality	Collaborative	An estimated 981 sepsis cases and 172 deaths were averted in the post-British Columbia Sepsis Network period (2014–2018). The total cost, including the development and implementation of British Columbia Sepsis Network, was $449,962. Net savings due to cases averted after program costs were considered were $50.6 million in 2018. This translates into a return of $112.5 for every dollar invested

### Process improvement approach

The scope of the PI implementation in the hospitals described in the seven papers was diverse. Three of the papers described a whole of hospital focussed PI approach while others incorporated sections of hospital care processes or specific patient populations in their studies All sufficiently described the PI methodology employed.

Two studies described a ‘value-driven’ outcomes approach whereby the PI initiatives were aimed at increasing the value of care provided where value is defined as health outcomes per dollar spent [18, 19]. A third article took an approach focussed on improving the quality outcomes of surgical care as measured by reduction in adverse events, recognising that this brings with it a concomitant financial impact that can be measured [20]. All three studies report using a "standardised process improvement model" or “Continuous Improvement” to deliver the improvements [18, 19]. A fourth study focussed on implementation of evidence-based sepsis management and used a recognised PI methodology coined by the Institute for Healthcare Improvement called Collaboratives.

The remaining three studies took a broad, whole of hospital improvement approach using Lean methodology and particularly focussed on removing unnecessary healthcare delivery processes and waste [21, 22]. Resultant financial impact as well as patient outcomes were measured and reported.

In summary, the seven papers identified in the search strategy indicate that PI is frequently used to reduce waste and improve the value of clinical care and processes of care in hospitals. The PI methods used in these studies varied, however there was implied consensus that a structured problem-solving methodology supports the achievement of improved clinical care and a reduction in non-value adding processes.

### Financial measurement and realization approach

All seven studies sought to include financial outcomes of PI activities in their methodologies in acknowledgement of, and to address the continuous increase in healthcare expenditure. Each study used different methods to calculate the financial impact of their PI activities with only two of the studies specifying whether there was a financial impact at the enterprise level. The following describes the different approaches taken by the hospitals to measure financial benefits.

One study ([19] p. 1062) developed their own tool called the ‘value-driven outcomes tool’ to *“…… (1) identify overall care costs across the health care system, (2) measure cost variability across Medicare severity diagnosis related groups (MS-DRGs) to identify the greatest opportunities for cost reduction and outcome optimization, and (3) support value improvement initiatives for selected conditions.”* In this study, direct patient costs only were included in the financial outcomes measured with two of the three improvement initiatives reporting a reduction in direct patient costs and the financial outcome of the third project not reported. There was no evidence provided as to the quantum (dollar value) of these savings or whether these savings resulted in an impact at the hospital enterprise level.

Another paper reported savings of $1.3 million dollars over the study period of 2009–2015, after the input costs of membership of the American College of Surgeons National Surgical Quality Improvement Program (ACS NSQIP) were deducted [20]. The cost savings were based on a reduction in six types of adverse events from clinical care improvements implemented for all surgical cases across the six year period. The savings were estimated using a cost calculator provided by the ACS NSQIP with no further detail of the financial measurement methodology available. This study took the input costs (the cost of ACS NSQIP membership) into account in the measurement methodology. The paper also doesn’t declare whether these savings were realized or detectable at the enterprise level.

The third paper undertook 74 small clinical improvement initiatives spanning the whole hospital over a three year period, with the intention of increasing the value of the care provided [18]. The hospital used a cost-accounting system to also measure only the direct costs of acute patient care before and after the PI interventions. This paper identifies the financial benefits of the PI activities as *“…… a 7.7% drop in mean monthly variable direct cost per case* and also notes the resultant financial impact at the enterprise level as “….. *cutting institutional expenses by $59.3 million”* ([18] p455)*.*

The fourth study was also a whole of hospital PI (Lean) implementation that focussed on removing waste and non-value adding processes. Over the seven years that the study covers, the hospital estimated the cumulative realized financial benefit to be nearly $200 million dollars with concurrent improvements in key quality indicators such as mortality and staff engagement. The study provides some explanation of how these financial measures were calculated including “…… *savings (dollars that did not go out the door) and two measures of revenue increases: increased productivity with the same resources or increased revenue accrued from a new or Lean-improved process”* ([21] p. 125)*.* The paper also refers to ‘hard green dollars’ and that unless savings resulting in staff time being ‘released’ could be translated into actual staff number reductions, then the savings would not count towards the total financial impact. Similarly, if costs were avoided through process redesign, they would not ‘count’. Further detailed financial methodology is not included in the paper however it is implied that these savings impacted at the enterprise level. Further detailed financial methodology is not included in the paper however it is implied that these savings impacted at the enterprise level.

The fifth paper provided an *‘economic’* impact from one of the five streams of work (surgery) purporting to have achieved saving of 8,563,817 € through reducing waste and reordering surgical schedule and a revenue of increase of 600,044 € due to increasing surgical activity [23]. There was no reference to the capture of these financial benefits and impact on the enterprise budget.

The sixth paper alluded to *‘financial stewardship’* measures noting that 11 (48%) of the 23 financial stewardship drivers improved in 2010 compared to 2009. Further financial information from 3 specific improvement initiatives were noted with financial benefits identified including average decrease in cost per case of 22%, 895,000 in salary savings increased gross revenue by 24%. No financial calculation methods were described including at an enterprise level.

The final paper [24] used a cost estimate per sepsis case or death due to sepsis averted over the study period. The costs were estimated as the difference between the cost of managing a case of sepsis in British Columbia hospitals and the cost of standard hospital stay for a patient without the diagnosis of sepsis calculated from an average during the baseline period. Table 3 provides a summary of the financial outcomes reported in each paper.

**Table 3:** Financial outcomes and enterprise impact

Citation	Country	PI scope	PI financial outcomes reported?	PI Financial methods described?	Type of PI financial outcomes	Enterprise impact from PI specified?
Gabow, P. A. (2014). The Lean Prescription: Powerful Medicine for Our Ailing Healthcare System (1st ed.): OR: Productivity Press	U.S	Whole hospital	**√**	**X**	money not spent (‘savings’) revenue increases	**√**
Chatfield SC, Volpicelli FM, Adler NM, et al. Bending the cost curve: time series analysis of a value transformation programme at an academic medical centre BMJ Qual Saf 2019;28:449–458	U.S	Whole hospital	**√**	**X**	mean monthly variable direct cost per case	**√**
Barnas, K. (2011) ThedaCare’s Business Performance System: Sustaining Continuous Daily Improvement Through Hospital Management in a Lean Environment. The Joint Commission Journal on Quality and Patient Safety, 37( 9), 387–399	U.S	Whole hospital	**√**	**X**	increased revenue reduced staff costs costs per case	**X**
Lee, V. S., Kawamoto, K., Hess, R., Park, C., Young, J., Hunter, C., Pendleton, R. C. (2016). Implementation of a Value-Driven Outcomes Program to Identify High Variability in Clinical Costs and Outcomes and Association With Reduced Cost and Improved Quality. JAMA, 316(10), 1061–1072	U.S	3 Clinical projects	**√**	**√**	Reduced direct patient care costs	**X**
Nimeri, A. A., Bautista, J., & Philip, R. (2019). Reducing healthcare costs using ACS NSQIP-driven quality improvement projects: a success story from Sheikh Khalifa Medical City (SKMC). World journal of surgery, 43(2), 331–338	U.A.E	Surgical Value Stream	**√**	**X**	Money not spent (due to preventing adverse events)	**X**
Vincent Martinez Ibañez, Anna Ochoa de Echagüen, Antonio Campos & Soledad Romea (2021) Creating efficient professional healthcare organizations, International Journal of Healthcare Management, https://doi.org/10.1080/20479700.2020.1870347	Spain	5 value/process streams	**√**	**X**	increased revenue cost savings	**X**
Khowaja AR, Willms AJ, Krause C, Carriere S, Ridout B, Kennedy C, et al. The Return on Investment of a Province-Wide Quality Improvement Initiative for Reducing In-Hospital Sepsis Rates and Mortality in British Columbia, Canada. Critical Care Medicine. 2022;50(4):e340-e50	Canada	All in-hospital sepsis cases and sepsis mortality	**√**	**X**	Money not spent (due to preventing adverse events)	**X**

## Discussion

### Principal findings

The objective of this review was to answer two research questions. The first question sought to identify the methods that hospitals use to measure and realize the financial benefits of PI activities. The search identified many papers outlining the use of PI to reduce waste and improve the value of clinical care and processes of care in hospitals but only a small number that also report resultant financial benefits measurement and capture. Of those papers identified that report financial benefits measurement, the calculation methods differed, and the level of detail provided varied. This paucity of information and variation in method makes it challenging for other hospitals to use the published evidence in practice.

The second research question this paper is pursuing is whether hospitals collate the financial benefits from PI programs at the hospital enterprise level. The reason why this is important is that without this step, hospitals are not able to impact their overall spending and so contribute to reducing the cost burden of healthcare overall [25]. This review identifies that translating the financial impact of PI activity at the enterprise level is possible but is not consistently performed. The disconnect between the measurement of a financial benefit and its capture at the enterprise may arise for two possible reasons. The first is because of the high fixed costs inherent in hospitals that remain regardless of the quality of clinical care. The second is the nature of ‘light green dollars’ released through efficiency gains or improved productivity, which is an established outcome of PI programs [11]. Nolan and Bisognano ([26] p. 69) explain light green dollars as *“….. potential savings….”* as compared to dark green dollars, which they call *"…. real savings”.* The distinction between measurement of financial benefit at the PI activity level and the hospital or enterprise level is pertinent to the research question, for without the ability to not only measure but also capture the financial benefits of PI initiatives and program at a whole at the hospital level, it will be challenging to be able to impact the financial position of the system as a whole [27].

It is evident from this review, that there are different ways of thinking about cost savings in healthcare. That is, costs can be measured at the individual patient level, at a patient cohort level (i.e. orthopaedic surgical patients) or at the PI initiative level. What is not clear from the literature is which level of cost analysis, if any, is most pertinent in delivering a positive enterprise level financial outcome. Porter [28] centres his definition of value at the individual patient level, noting that it is the inability to define cost of an entire patient journey across the multiple organizational silos that exist, that inhibits the ability of a hospital to realize the impact of improvement efforts [28]. Perhaps one reason for this is that in healthcare, costing is difficult at the individual patient level, but somewhat easier at the department level hence the tendency for research to be at this level rather than the hospital enterprise level [19, 29].

There are many factors that impact the financial performance of a hospital, besides the volume of potential waste in the processes of care delivery, the underlying value of that care and the impact of its removal or reduction. Stock and McDermott [30] observe that reducing costs in hospitals is difficult and in fact merely calculating costs of care may be the biggest challenge. Martin et al. ([31] p. 1) suggest that hospitals have been challenged in capturing actual rather than theoretical savings resulting from PI efforts and that this may be because it is *“… rarely (if ever) possible to track the savings to a specific finances line item.”* Porter ([28] p. 2478) supports this stating that *“The current organizational structure and information systems of health care delivery make it challenging to measure (and deliver) value. Thus, most providers fail to do so.”* As such, the inherent difficulty in defining patient and hospital costs makes measuring and realising financial outcomes from PI challenging.

This review found that little is published regarding the capture and collation of the financial benefits of PI to the hospital enterprise budget. Hospitals may be applying the financial benefits of PI internally and keeping the methods confidential. However, a recent study by the authors [25] and others [26, 31] found that hospital managers admitted that this practice should be followed but they had difficulty. One potential reason is that the financial benefits are (and should be) an outcome of the effort, not the driver itself. Hospitals may deliberately avoid measuring financial benefits of PI so that healthcare professionals do not associate PI as a cost cutting exercise. However, the concept of value-based healthcare is now well accepted across health professions [32] and this brings with it the costs of care that are inherent in the definition (outcomes per dollar spent). Swensen et al. ([33] p. 534) call on their medical colleagues to take action around waste in hospital processes and medical care, suggesting some simple and immediate ways that they can act to reduce non-value adding process steps such as “…. *a physician-led reduction in (1) overuse of hospitals, (2) preventable complications and (3) waste within healthcare processes.”*

All studies included in the review were conducted between 2005 and 2018 (although were published up until 2022). This may indicate that as healthcare cost pressures continue to grow, there is increasing attention and effort on cost reduction. It may also indicate that as the application of PI in healthcare matures, hospitals have been able to build capability to include the financial outcomes of PI in their methodologies.

Four of the seven papers are from hospitals in the U.S. with the remaining three papers from a hospital based in the UAE, one in Canada and another in Spain. With significant financial pressure on hospitals in the U.S. given the high proportion of the GDP that is spent on their healthcare system, this may result in a greater will to identify and realize financial benefits from improving the value of care [34]. The U.S. healthcare system is largely private and is therefore different to others in the world in terms of the ability to invest in technology systems and people to support their PI programs. As such, when framing public health policy outside of the U.S., the need for investment in supporting systems, structures and people, to garner the financial outcomes manifest in PI initiatives to create value-based care must be considered.

Narayanan et al. (2021) note that lean implementation studies have generally found a positive correlation with revenue increases as the primary positive financial impact of PI. These studies have all been in the U.S., where increased efficiency and throughput is likely to be associated with ’doing more work’ which in turn generates more income. In public hospitals in countries like Australia, increased efficiency results in more patients being treated within the same funding bucket. It is proposed however, that the key concepts of PI as the means by which waste is minimized and high value care is maximised with resultant financial benefits able to be measured and captured are relevant to other healthcare sectors and geographic contexts.

Rauh et al. [35] describe the belief of many that by improving the quality of care delivered, the costs will follow resulting in reduced health care costs. They note that after many decades of healthcare improvement efforts around the world, these reduced costs from clinical care improvements rarely materialise. They explain this by suggesting that the rigid healthcare and financial structures generate improved efficiencies through improving clinical care, rather than bottom line savings. Porter supports this noting that *“The current organizational structure and information systems of health care delivery make it challenging to measure (and deliver) value. Thus, most providers fail to do so.”*

### Practice implications

Two of the papers’ PI efforts were explicitly targeted at increasing the value of care delivered. As Porter’s definition of value incorporates both quality and cost outcomes and as hospitals strive to increase value and reduce expenditure, they must necessarily also pay attention to the financial outcomes of PI. Hospitals measure patient outcomes (quality and experience) at the individual level but are rarely able to match the cost component at this level with cost accounting systems not being available, fit for this purpose or used. As such, further investment in patient level costing systems may support hospitals to better measure financial benefits arising from PI, and then capture and collate them.

Hofer et al. (2010) note that the relationship between PI and financial benefits is likely to be complex and multifaceted and that the exact mechanism(s) through which they are linked is not well researched [36]. This is supported by the dearth of papers identified by this review and is perhaps a call to action for those hospitals who are currently measuring financial benefits of PI to publish their methods and outcomes for other hospitals to learn from.

Hospitals should be working to embed a PI methodology that incorporates both quality and financial benefits realization as a core component of their operational management systems. Inclusion of staff with financial literacy and expertise in PI programs may also support routine financial benefits measurement. In doing so, hospitals will be supporting broader efforts to reduce the cost burden of healthcare on governments, funders, and healthcare users.

From a policy perspective, governments have the opportunity to support hospitals to increase high value care, reduce or remove low value care and reduce process waste with investments in necessary infrastructure such as clinical costing and accounting systems. Investment in PI staff and programs to support clinicians to lead PI initiatives along with specialists to support financial benefits realization is also required. Governments could consider ensuring funding policies are aligned to supporting the provision of high value clinical care but this is perhaps a longer term objective, as proposed by Duckett et al. [2].

Finally, the OECD [1] puts forward the simple justification that it is not easy for those responsible for the cost of healthcare (governments, hospital administrators and clinicians) to concede that such a large percentage of what is spent does not add value to the patient. This may be the single biggest contributing factor to the dearth of research published on this topic.

### Strengths and limitations

While a broad net was cast for the search, it is possible that the search terms and the databases searched were not sufficiently sensitive or specific enough to capture all relevant studies that considered both PI and financial outcome measurement and capture. With only seven papers included in the review, this may limit the validity of the findings and policy recommendations. This is not entirely unexpected with D’Andreamatteo et al. [37] and Narayanan et al. [13] both calling for further research to be conducted in the healthcare sector in relation to the financial outcomes of PI following their literature reviews.

The GRADE approach to rating the quality of evidence included in a systematic review has been employed to reflect the confidence in the findings and recommendations [17]. The GRADE approach specifies four categories that can be applied to a body of evidence rather than to individual studies: high; moderate; low; and very low. In applying the GRADE approach to this study, the body of evidence identified through the systematic review is categorised as moderate and indicates that the authors of the review are *“….. moderately confident in the effect estimate: The true effect is likely to be close to the estimate of the effect, but there is a possibility that it is substantially different.” *([17] p. 404)*.*

Another limitation with the review is that all papers included in the study reported a positive financial outcome of their PI initiatives which while intuitively would be expected when the value of care is improved, it may reflect possible publication bias.

## Conclusion

Evidence has been mounting that the costs of healthcare are too high and unsustainable. One of the causes is the volume of clinical and care activities and processes that do not add value to outcomes or experience.

This study demonstrates that hospitals can measure positive financial outcomes from PI initiatives, but that it is difficult to capture the benefits at the enterprise level. The papers included in the review suggest that the impetus for the PI initiatives where financial benefits are measured, is driven by the pursuit of an increase in high value clinical care provision and a reduction of waste in processes. Finally, the study also demonstrates that there is very little literature published in the field of PI and enterprise financial benefits measurement in healthcare.

The financial measurement methods used vary in terms of cost inclusions and exclusions and the ‘level’ at which the costs were measured (i.e. patient level or PI initiative level) making comparisons difficult. What is clear however, is that to impact expenditure, hospitals must be intentional in finding a method to measure and capture financial benefits alongside other PI outcomes. Further research on best practice financial measurement methods could support other hospitals to include explicit realization of financial benefits in their standard PI methodology.

## Data Availability

The datasets used and/or analysed during the current study available from the corresponding author on reasonable request.
